# Sleep disturbance and its associated factors among pregnant women in Ethiopia: systematic review and meta-analysis

**DOI:** 10.1186/s12888-023-05456-7

**Published:** 2024-01-15

**Authors:** Sintayehu Simie Tsega, Mekdes Kiflu, Sisay Maru Wubante, Birye Dessalegn Mekonnen, Yeshambel Andargie Tarekegn

**Affiliations:** 1 Department of Medical Nursing, School of Nursing, College of Medicine and Health Science, University of Gondar, Gondar, Ethiopia; 2 Clinical pharmacy unit, Department of Pharmacy, College of Health Science, Debre Markos University, Debre Markos, Ethiopia; 3 Department of Health Informatics, Institute of Public Health, College of Medicine and Health Science, University of Gondar, Gondar, Ethiopia; 4 Amhara Public Health Institute, Bahir Dar, Ethiopia; 5 Department of Otorhinolaryngology (ENT), School of Medicine, College of Medicine and Health Science, University of Gondar, Gondar, Ethiopia

**Keywords:** Sleep disturbance, Associated factors, Pregnant women, Ethiopia

## Abstract

**Introduction:**

Globally, sleep disturbance is the foremost public health issue among pregnant women which might have undesirable birth outcome including neurocognitive impairment, preterm birth, low birth weight, and neonatal morbidity and mortality. In Ethiopia, inconsistent findings have been reported on the prevalence of sleep disturbance among pregnant women. Therefore, this review aims to estimate the pooled prevalence sleep disturbance and its associated factors among pregnant women in Ethiopia.

**Methods:**

This systematic review and meta-analysis of observational studies was designed according to the PRISMA guideline. A systematic search of literature was conducted in PubMed, Scopus, Web of science, and Google Scholar using relevant searching key terms. The Newcastle-Ottawa scale was used to evaluate the quality of all selected articles. Data were analyzed using STATA Version 14 software. Publication bias was checked using Egger’s test and funnel plot. Cochran’s chi-squared test and I^2^ values were used to assess heterogeneity. A fixed-effects model was applied during meta-analysis.

**Results:**

In this review, six studies were included after reviewing 17,100 articles. The pooled prevalence of sleep disturbance among pregnant women in Ethiopia was 50.43% (95%CI: 39.34–61.52). Third trimester pregnancy AOR = 4.03; 95% CI: 2.84,5.71), multigravidity (AOR = 1.99; 95% CI: 1.54, 2.59), unplanned pregnancy (AOR = 2.56; 95% CI: 1.52,4.31), depression (AOR = 3.57; 95% CI: 2.04, 6.27), stress (AOR = 2.77; 95% CI: 1.57, 4.88), anxiety (AOR = 3.69; 95% CI: 1.42, 9.59) and poor sleep hygiene (AOR = 2.49; 95% CI: 1.56, 3.99) and were statistically associated with sleep disturbance among pregnant women.

**Conclusion:**

This review revealed that the magnitude of sleep disturbance among pregnant woman in Ethiopia was relatively high and multiple factors determined the likelihood of having a disturbed sleep-awake pattern. Thus, the implementation of interventions for sleep disturbance after screening pregnant women is needed. Moreover, public health interventions targeted on the prevention of unintended pregnancy and depression during pregnancy should be implemented.

## Introduction

Sleep disturbance is a change of sleeping habits in quality and/ or quantity of sleep [1]. It encompasses disorders of initiating and maintaining sleep, excessive daytime sleepiness, and, disorders of sleep-wake cycle [2, 3]. Sleep disturbance is a critical public health concern that affects the overall productivity of the country by decreasing an individual’s ability to comprehend and accomplish their day-to-day lives, disrupt school or work performance, and diminishing mental and physical health [4, 5].

During pregnancy, the inconsistent sleep awake cycle in an indicator of sleep disturbance [6–9] they have increased, normal and decreased sleep pattern in their total sleep time in the first trimester, second and third trimester of pregnancy respectively [3, 9, 10]. Sleep disturbance is highly predominant during pregnancy and are commonly overlooked as a potential cause of maternal and fetal morbidity [3, 11]. Women’s sleep-awake cycle is frequently disturbed during their pregnancy and it can diminishes the usual health functioning [9, 12] and increases pregnancy related psychiatric comorbidities including anxiety and depression [11, 13]. As the gestational age of pregnancy increases, the more likely women to have frequent awakenings and inadequate sleep habits [14, 15].

Sleep disturbance causes maternal and fetal mental impairment [16], preterm birth [17], low birth weight [11, 18], increase the risk of developing gestational diabetes mellitus [19–21] and the offspring predisposed to developmental delay and learning disabilities [22].

Sleep disturbance among pregnant women can be due to the hormonal and mental changes that the body undergoes during pregnancy time [23]. This may be due to mechanical and physical changes which leads the women to experience discomforts including leg cramps, urinary incontinence, and shortness of breath, and intense backaches [24]. Marked increment in the level of estrogen and progesterone because of the pregnancy influences a diverse range of both physiological and psychological processes, including sleep and mood disturbance [25] estrogen and oxytocin causes difficulty in breathing and sleep fragmentation [26].

Previous studies have identified multiple factors influence sleep disturbance among pregnant women such as unplanned pregnancy, third trimester anxiety depression and stress.

In Ethiopia, several individual studies have been conducted on the prevalence and factors of sleep disturbance among pregnant women. However, the findings are inconsistently reported from 30.8 to 68.4% and are not systematically reviewed. Therefore, the purpose of this systematic review and meta-analysis was to determine the pooled prevalence of sleep disturbance and its associated factors among Ethiopian pregnant women. The results of this systematic review and meta-analysis might help stakeholders and policymakers to implement different programs and healthcare initiatives aimed improving sleep quality and quantity of pregnant women.

## Method

### Information sources and searching strategy

All potential articles were retrieved from the electronic databases such as PubMed, EMBASE, Web of Sciences, and Google Scholar. Article searching was conducted using free text search terms and Medical Subject Headings (MeSH). We used the following searching terms: “Sleep disturbance,” “Poor sleep quality,” “fragmented sleep,” “Disturbed sleep awake cycle,” “pregnant women,” “Predictors,” “Factors,” “Risk factors,” “Prevalence,” “Proportion,” and “Ethiopia.” The search was conducted using Boolean operators like “AND” and “OR” and truncations without publication date restriction. Articles were searched from October 12 to November 28, 2023. For PubMed searching we have used this searching formulation: ((sleep disturbance*[All Fields]) OR (poor sleep quality*[All Fields])) OR (fragmented sleep*[All Fields])) OR (disturbed sleep awake cycle*[All Fields])) AND (Pregnant mothers*[All Fields])) OR (women attending antenatal care follow-up*[All Fields])) AND (predictors) OR (risk factors[MeSH Terms]) OR (associated factors) OR (Barriers [MeSH Terms]) AND (Prevalence) OR (Proportion[MeSH Terms]) AND (Ethiopia)).The review was done following the Preferred Reporting Items for Systematic Reviews and Meta-Analyses (PRISMA) checklist [27].

#### Inclusion and exclusion criteria

Observational studies focusing primarily on sleep disturbance, articles assessing factors related to sleep disturbance among pregnant women and conducted in any study period (the study period was not restricted for inclusion),were some of the inclusion criteria of these review. Whereas, editorial letters, reviews and commentaries were excluded from this review. Finally, reviewers independently examined the research’s eligibility, and any discrepancies were handled by discussion and consensus.

#### Study selection

Initially, all the retrieved articles were exported to EndNote X7 reference manager software to manage duplicate studies, and the screening process. Then, two review authors (SST, YAT) independently screened articles by their titles and abstracts after the exclusion of duplicates. Consecutively, the full text of potentially eligible articles were retrieved and screened using predetermined inclusion and exclusion criteria. The disagreements between authors during study screening were solved by the remaining review authors (MK, SMW, and BDM).

##### Exposure

Factors or determinants of sleep disturbance.

##### Outcome

Pregnant women have experienced sleep disturbance.

#### Outcome measurement

The primary outcome of this review was the prevalence of sleep disturbance among pregnant women. It was assessed by the Pittsburgh Sleep Quality Index (PSQI), a Self-report questioner containing 19 items assessing seven components of sleep: subjective sleep quality, sleep latency, sleep duration, habitual sleep efficiency, sleep disturbances, daytime dysfunction, and use of sleep medications. Each component is scored (range 0–3). A total global PSQI score ranges from 0 to 21, a global score of ≥ 5 was classified as pregnant women having sleep disturbance. The second outcome of this review was determining the factors associated with sleep disturbance among pregnant women in Ethiopia and were measured with the odds ratio (OR). The odds ratio was calculated for each identified factor based on the binary result data presented by each study.

### Quality assessment

To measure the quality of each original studies, the Newcastle-Ottawa Scale (NOS) tool has been used. The evaluation framework is divided into 3 areas: The first part of the method is a five-star rating system that evaluates the selection of study groups in each study. The second section evaluates the comparability of study with the likelihood of gaining two stars. The final part of the tool evaluates the appropriateness of statistical analysis that each primary study used, with three stars plausible. Subsequently, studies that scored > 6 stars out of 10 were considered as high quality 5 or 6 out of 10 were considered as good quality, and less than 5 were considered as poor quality. There were no studies excluded due to poor quality. Two review authors (SST, YAT) independently assessed the quality of studies with any disagreements were solved by the remaining review authors (MK, SMW, BDM).

#### Data extraction

To extract data from articles included in the review, a standardized data extraction tool was adapted from the Joanna Briggs Institute (JBI) was used. Two authors (SST and YAT) independently extracted the data from each primary study included in this meta-analysis. From each study information such as the first author’s name, the study’s region and setting, the year of publication, the study design, the study participants, the sampling technique, and the sample size, prevalence and factors associated with sleep disturbance, and measures of association (OR) were extracted.

### Statistical analysis

The extracted data were imported into STATA version 14 for analysis. Tables, figures, and forest plots were used to describe and summarize findings. We calculated the I^2^ statistic to determine study homogeneity, which describes the percentage of total variation among studies, in which 25%, 50%, and 75% represented low, moderate, and high heterogeneity, respectively [28]. A fixed-effects model was used to execute the pooled estimate of sleep disturbance and to calculate the pooled OR for each identified factors if substantial heterogeneity was observed, otherwise, a random-effects model was used. Furthermore, a graphic review of the funnel plot and an Egger’s regression test were used to determine the presence or absence of publication bias. However, publication bias for each factors was not evaluated due to limited number of studies.

### Publication bias

**Fig. 1:**
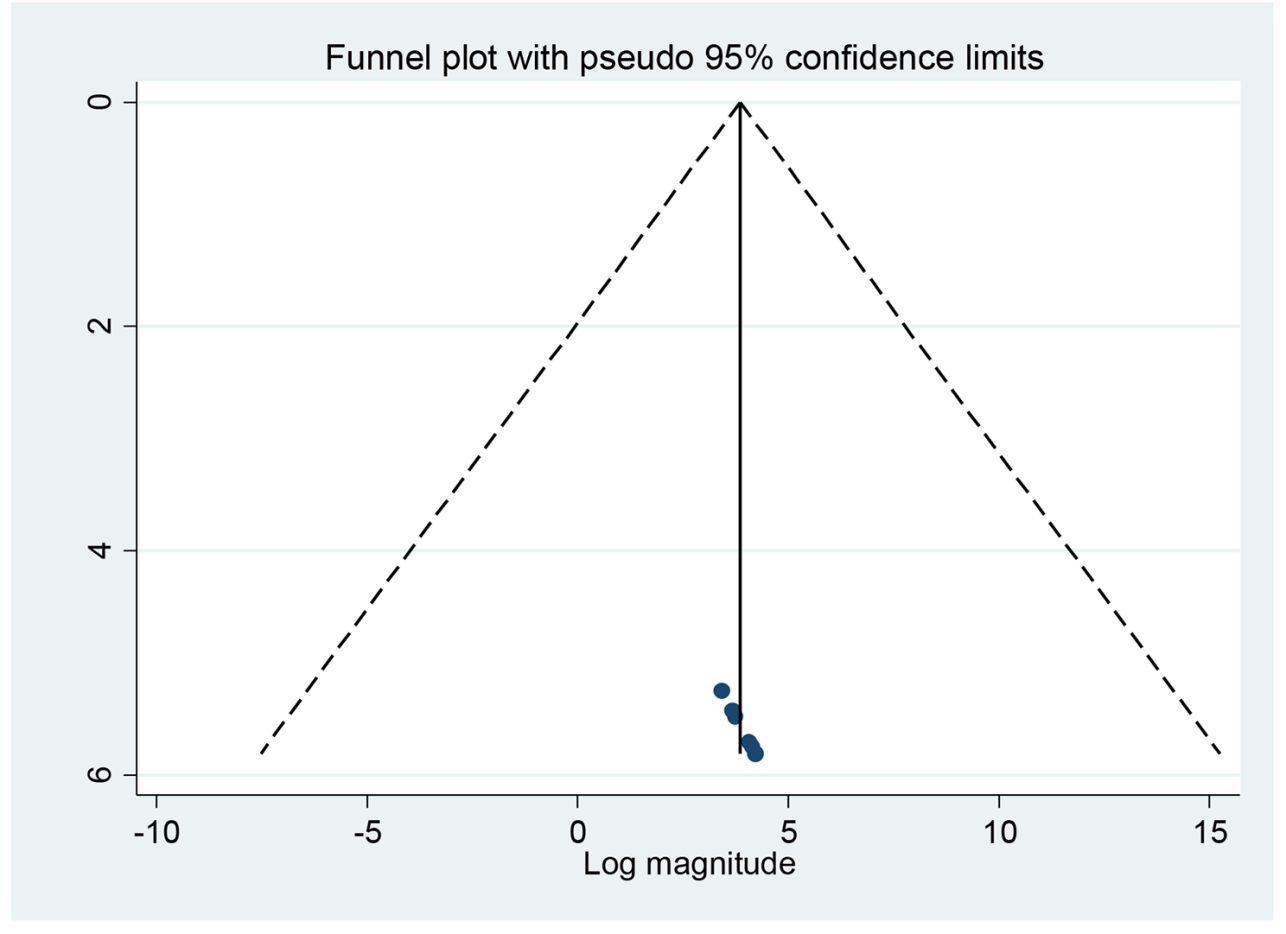
Publication bias detection using Funnel plot on sleep disturbance among pregnant women in Ethiopia 2023

A graphic review of the funnel plot and an Egger’s regression test were used to determine the presence or absence of publication bias. Accordingly, the results of the funnel plots and Egger’s regression test in this meta-analysis showed that there is no evidence of publication bias (Fig. 1).

## Result

### Searching results

**Fig. 2:**
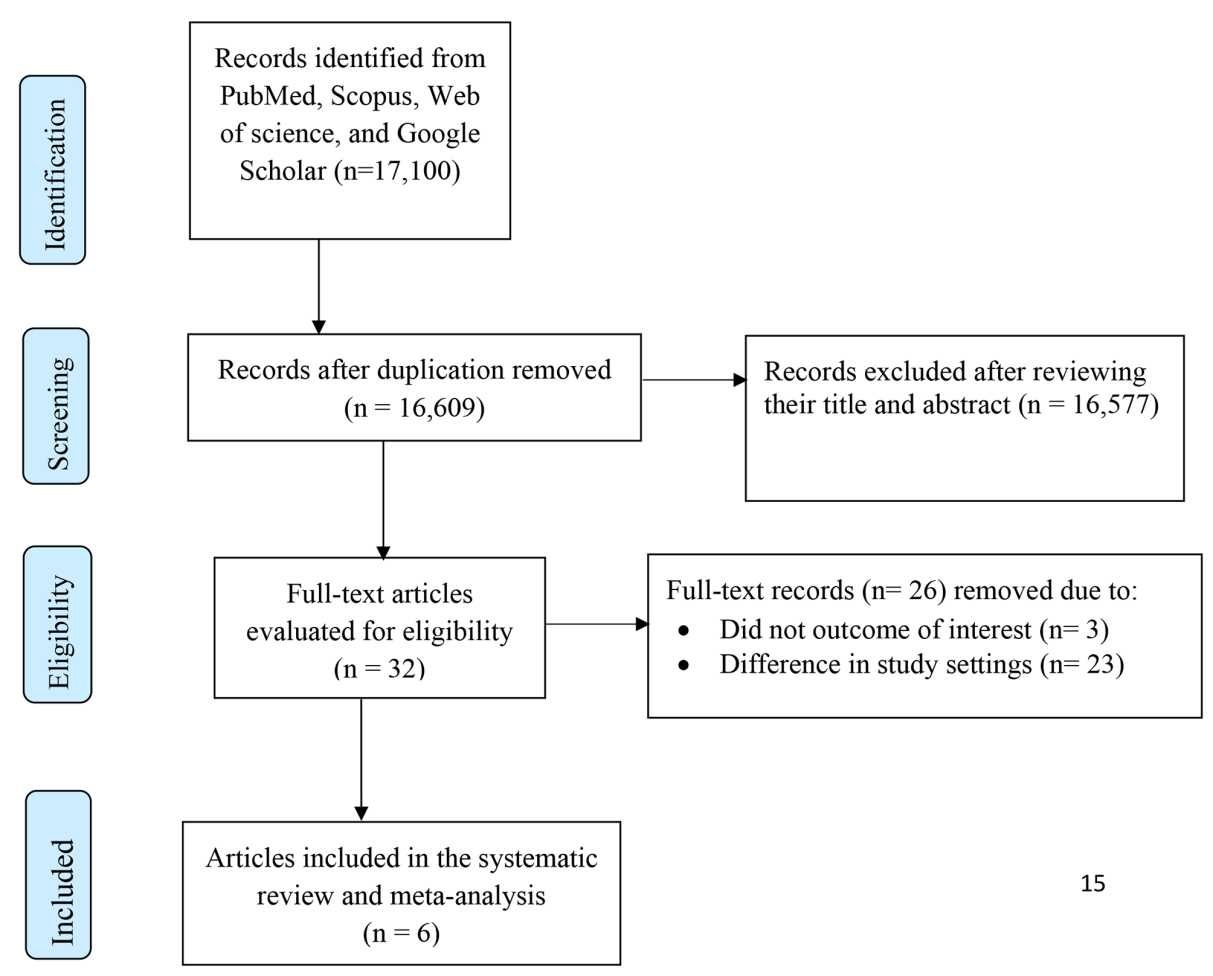
PRISMA flow diagram for meta-analysis of sleep disturbance and its associated factors among pregnant women in Ethiopia

A total of 17,100 articles were retrieved from different electronic databases, as well as from Google Scholar. About 491 duplicate articles were removed using EndNote citation manager; 16,577 articles were excluded after the title and abstract screening, and then 32 articles remained. After a careful review of the articles for the presence of the outcome variable and other inclusion criteria, 26 papers were removed in compliance with the exclusion criteria. Finally, six articles remained for the analysis. The overall study selection process was represented by the following flow diagram. (Fig. 2).

### Characteristics of the included studies

All the included studies used a facility-based cross-sectional study design and conducted from 2019 to 2020. One study included in this review employed simple random sampling [29], whereas the others used systematic random sampling [30–34]. All studies employed an interviewer-administered sampling method to choose the study participants. A total of 2483 pregnant women took part in the review. According to the research examined, sleep disturbance among pregnant women ranged from 30.8% [30] to 68.4% [31]. Regarding study region, three studies were done in Amhara, two studies in Oromia, and one study were conducted in multiple regions, Northwest Ethiopia (Table 1).

**Table 1:** prevalence of poor sleep quality according to selected variables

Author	Region/study area	Study Year	study population	Sample Size	Prevalence
Anbesaw et al. [30]	Oromia	2020	Pregnant women	415	30.8
Jemere et al. [31]	Amhara	2020	Pregnant women	411	68.4
Takelle et al. [32]	Amhara	2020	Pregnant women	415	42.2
Amare et al. [49]	Amhara	2020	Pregnant women	423	62.8
Tasisa et al. [34]	Oromia	2019	Pregnant women	408	59.1
Legas et al. [29]	Northwest Ethiopia	2020	HIV-positive pregnant women	411	39.4

### Prevalence of sleep disturbance among pregnant women in Ethiopia

**Fig. 3:**
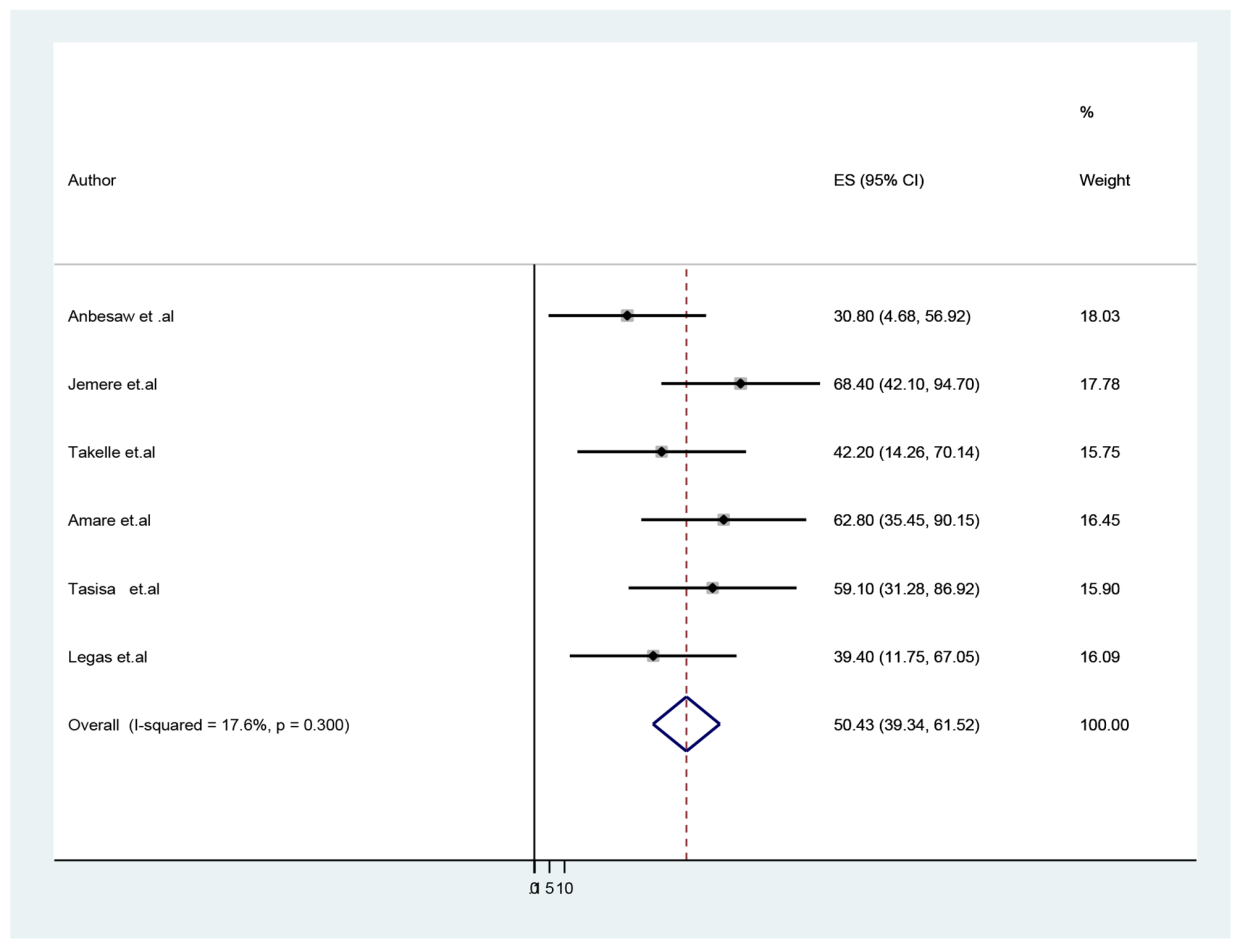
Forest plot showing the pooled prevalence of sleep disturbance among pregnant women in Ethiopia 2023

The results of this meta-analysis showed that the overall pooled prevalence of sleep disturbance among pregnant women in Ethiopia was 50.43% (95%CI: 39.34–61.52). During this meta-analysis, a fixed-effects model was applied as no heterogeneity was observed among the included studies (Fig. 3).

### Factors associated with sleep disturbance among pregnant women

In this review, depression, stress, third trimester pregnancy, anxiety, unplanned pregnancy, poor sleep hygiene and multigravidity were the factors associated with sleep disturbance among pregnant women in Ethiopia.

Meta-analysis of four studies showed that third trimester pregnancy has a significant association with sleep disturbance. The pooled odds of sleep disturbance were more than four times (AOR = 4.03; 95% CI: 2.84, 5.71) higher in pregnant women during third trimester pregnancy as compared to first and second trimester. Two studies showed that multigravidity has a significant association with sleep disturbance. The overall odds of sleep disturbance were about 1.99 times (AOR = 1.99; 95% CI: 1.54, 2.59) higher among prim gravid pregnant women. Two studies showed that unplanned pregnancy has a significant association with sleep disturbance. The overall odds of sleep disturbance were 2.56 times (AOR = 2.56; 95% CI: 1.52, 4.31) higher among pregnant women who had unplanned pregnancy than their counterparts. Three studies showed that pregnant women who had depression during pregnancy were 3.57 times (AOR = 3.57; 95% CI: 2.04, 6.27) more likely to experience sleep disturbance than their counterparts. Three studies also showed that stress has a significant association with sleep disturbance among pregnant women. The overall odds of sleep disturbance were 2.77 times (AOR = 2.77; 95% CI: 1.57, 4.88) higher among pregnant women who had stress than their counterparts. Two studies indicated that anxiety has an association with disturbed sleep. The pooled odds of sleep disturbance were about 3.69 times (AOR = 3.69; 95% CI: 1.42, 9.59) higher among pregnant women with anxiety than their counterparts. Two studies indicated that poor sleep hygiene has an association with sleep disturbance. The pooled odds of sleep disturbance were about 2.49 times (AOR = 2.49; 95% CI: 1.56, 3.99) higher among pregnant women who have poor sleep hygiene practice than their counterparts. (Table 2).

**Table 2:** Factors associated with sleep disturbance among pregnant women in Ethiopia 2023

Factors associated with sleep disturbance	Studies	OR (95% CI)	Pooled OR % (95% CI)	Test of heterogeneity	
				I^2^	P
Third trimester pregnancy	Jemere et al. Takelle et al. Amare et al. Tasisa et al.	7.50 (3.18, 17.69) 3.45 (2.05,5.80) 2.51 (1.17,5.40) 5.84 (2.54,13.45)	4.03 (2.84,5.71)	34.5	0.205
Multigravidity	Anbesaw et al. Amare et al.	1.90 (1.44,2.51) 2.72 (1.34,5.51)	1.99 (1.54,2.59)	0.00	0.354
Unplanned pregnancy	Tasisa et al. Legas et al.	4.25 (1.47,12.26) 2.18 (1.20,3.96)	2.56 (1.52,4.31)	13.6	0.282
Depression	Anbesaw et al. Takelle et al. Tasisa et al.	4.26 (2.54,7.14) 2.12 (1.19,3.77) 5.73 (2.49,13.20)	3.57 (2.04,6.27)	58.4	0.090
Stress	Anbesaw et al. Takelle et al. Legas et al.	1.85 (1.17,2.93) 5.39 (1.96,14.81) 3.10 (1.60,6.01)	2.77 (1.57, 4.88)	52.9	0.120
Anxiety	Tasisa et al. Legas et al.	6.62 (2.61,16.81) 2.46 (1.58,3.82)	3.69 (1.42,9.59)	71.8	0.060
Poor sleep hygiene practice	Legas et al. Tasisa et al.	2.23 (1.21,4.10) 2.93 (1.41,6.09)	2.49 (1.56,3.99)	0.00	0.574

## Discussion

This systematic review and meta-analysis was employed to estimate the pooled prevalence of sleep disturbance and predictors among pregnant women in Ethiopia. As a result, half of pregnant women in Ethiopia have a disturbed sleep pattern. This finding underlines the significance of timely screening, early diagnosis and providing proper intervention of sleep disturbance among pregnant women.

The pooled estimate of sleep disturbance among pregnant women in Ethiopia was 50.43%, (95% CI: 38.21–62.65). This study finding is in line with previous studies 45.7% [35] and 54.2% [36]. However, the results of this meta-analysis were lower than a systematic review and met analytic study conducted on association between sleep disorder during pregnancy time and risk of postpartum depression in China76% [37].

The possible reasons for this variation might be due to the difference in the socioeconomic status of the study participants including the difference in the prevalence of prenatal depression and stress.

This review identified that third trimester pregnancy, multigravidity, unplanned pregnancy, depression, stress, anxiety, and poor sleep hygiene were the factors statistically associated with sleep disturbance among pregnant women in Ethiopia.

This research found an association between third trimester pregnancy and sleep disturbance. This finding is supported by previous reviews [3, 35, 37]. The possible reasons of sleep disturbance during the third trimester might be due to the physiologic changes because of the pregnancy including urinary frequency, fetal movement, lower back pain, leg cramps, heartburn, easily fatigability and abdominal discomfort [14]. Furthermore, when the pregnant woman approaches to her expected date of delivery, she might worry about the mode of delivery, labor, birth outcome and financial issues which all could negatively affect the sleep pattern.

Multigravidity has a significant association with sleep disturbance. This may be explained by the fact that maternal sleep quality is disturbed as a result of being overstressed about having extra roles after childbirth and the way they incorporate the new role and responsibilities as a mother. In addition, multigravid pregnant mothers complained that their sleep pattern is depend on their children’s sleep awake cycle. If children frequently wake up at night, mothers will have a disturbed sleep pattern.

The review also showed that unplanned pregnancy has a significant association with sleep disturbance which is consistent with another study [38]. This may be due to inadequate preparation for pregnancy and childbirth leading mothers to feel stressed with all the changes and challenges.

The odds of sleep disturbance were about more than three times greater among pregnant women with depression when compared with their counterparts. Another study supports this finding [39]. This could be due to mood and emotional disturbance results sleep disturbance as depression and sleep disturbance have a bidirectional relationship [40]. Furthermore, evidence has indicated that prenatal depression is one of the most possible psychological factors contributing to sleep disturbance during pregnancy [9, 39].

This review also showed that stress has an association with sleep disturbance which is supported by previous studies [41, 42]. These may be due to stress is thought to increase cognitive and somatic arousal which negatively affects sleep primarily by decreasing sleep duration and results pregnant women to have a fragmented sleep pattern [43]. Moreover, it could be due to the direct effect of stress during pregnancy on sleep quality might be related to arginine vasopressin hormone, which is involved in the stress response and circadian regulation of the sleep-wake cycle [44, 45].

The findings of this review showed that anxiety has a significant association with sleep disturbance which is consentient with another study [46]. The odds of sleep disturbance were more than three times higher among pregnant women with anxiety as compared with their counter parts. The possible reason might be due to emotional and physiological arousal caused by anxiety and worries, which would result in more attention to environmental and personal stimuli, and these can lead to experience sleep disturbance [47, 48].

Furthermore, poor sleep hygiene practice has a significant association with sleep disturbance among pregnant women which is supported by another previous studies [3, 12]. The possible justification for this might be lack of healthy sleep habits, behaviors and environmental factors that can help pregnant women to have adequate sleep for instance drinking caffeinated drinks, performing dynamic physical activity and inconsistent sleep awake time.

### Limitation of the study

Even though this is the first systematic review and meta-analysis of sleep disturbance among pregnant women in Ethiopia, it is not without limitations. This review may not be representative for all regions as the included studies were done in some regions of Ethiopia. In addition, the causal association between outcome variable and factors couldn’t be established since all the included studies were cross-sectional in nature. As a result of limited number of studies, publication bias and subgroup analysis were not performed for each identified factors though heterogeneity was observed in some analyses.

### Conclusion and recommendation

This systematic review and meta-analysis found that about half of pregnant women have disturbed sleep pattern. Third trimester pregnancy, multigravidity, unplanned pregnancy, depression, stress, anxiety, and poor sleep hygiene were identified factors statistically associated with sleep disturbance. Thus, implementation of interventions for sleep disturbance after screening pregnant women is needed with collaborative effort of policy-makers and stakeholders. Moreover, public health interventions targeted at the prevention of unintended pregnancy, depression during pregnancy and considering other identified risk factors should be implemented.

## Data Availability

All data generated or analyzed during this study are available from the corresponding author with a reasonable request.
